# 

**Published:** 1991-03

**Authors:** 


South West Orthopaedic Club

South West Paediatric Club

South West Radiologists' Association

South West Urologists' Club

### WEST OF ENGLAND MEDICAL JOURNAL

**MARCH 1991** **Volume 106 (i)**

Copyright © West of England Medical Journal Ltd 1991.

**Contents**

2 **What Can We Do to Improve Relations between Clinicians and Managers?**
Peter Sims
5 **Twenty Years of Investigating Angiotensin Activity: an Overview**
Edward C. Osborn, J. Campbell Mackenzie and Linda M. Fearn
7 **Atlanto-axial Instability in Adults with Down's Syndrome**
Leila B. Cooke and R. Lansdall-Welfare
9 **Maintenance Dialysis in a District General Hospital**
Richard A. Banks and Jackie Folley
10 **From our Correspondents —**
A Lousy Story, Unauthorized Version
11 **References for Medical Posts – How Best to Utilise them**
H.K. Bourns
13 **The Water-Ram of Bristol and Neonatal Insufflation**
Peter M. Dunn
15 **Hydration Monitoring in the Long-term Prophylaxis of Nephrocalcinosis**
N.M. Goble
16 **Royal College of Surgeons Council Visit to Bristol**
17 **Bedding Out**
J.L. Burton
18 **South West Orthopaedic Club, November 1990 Meeting**
20 **South West Radiologists' Association, October 1990 Meeting**
21 **South West Urologists' Club, May 1990 Meeting**
23 **Wessex Branch of the Association of Clinical Pathologists, December 1990 Meeting**
27 **Obituaries —**
Mr. Ralph Arthur John Baily
Dr. Kenneth Harvey Gaskell
Mr. Gerald Newton Golden

***Scire est nescire, nisi id me alius sciret.*** **Lucilius (180–102 BC)**

